# An Artificial Intelligence-Based Melt Flow Rate Prediction Method for Analyzing Polymer Properties

**DOI:** 10.3390/polym17172382

**Published:** 2025-08-31

**Authors:** Mohammad Anwar Parvez, Ibrahim M. Mehedi

**Affiliations:** 1 Department of Chemical Engineering, College of Engineering, King Faisal University, Al-Ahsa 31982, Saudi Arabia; 2 School of Robotics, XJTLU Entrepreneur College (Taicang), Xi’an Jiaotong-Liverpool University, No. 111 Taicang Ave., Suzhou 215400, China

**Keywords:** artificial intelligence, machine learning, melt flow rate prediction, polymer properties analysis, pelican optimization algorithm

## Abstract

The polymer industry gained increasing importance due to the ability of polymers to replace traditional materials such as wood, glass, and metals in various applications, offering advantages such as high strength-to-weight ratio, corrosion resistance, and ease of fabrication. Among key performance indicators, melt flow rate (MFR) plays a crucial role in determining polymer quality and processability. However, conventional offline laboratory methods for measuring MFR are time-consuming and unsuitable for real-time quality control in industrial settings. To address this challenge, the study proposes a leveraging artificial intelligence with machine learning-based melt flow rate prediction for polymer properties analysis (LAIML-MFRPPPA) model. A dataset of 1044 polymer samples was used, incorporating six input features such as reactor temperature, pressure, hydrogen-to-propylene ratio, and catalyst feed rate, with MFR as the target variable. The input features were normalized using min–max scaling. Two ensemble models—kernel extreme learning machine (KELM) and random vector functional link (RVFL)—were developed and optimized using the pelican optimization algorithm (POA) for improved predictive accuracy. The proposed method outperformed traditional and deep learning models, achieving an R^2^ of 0.965, MAE of 0.09, RMSE of 0.12, and MAPE of 3.4%. A SHAP-based sensitivity analysis was conducted to interpret the influence of input features, confirming the dominance of melt temperature and molecular weight. Overall, the LAIML-MFRPPPA model offers a robust, accurate, and deployable solution for real-time polymer quality monitoring in manufacturing environments.

## 1. Introduction

A polymer is nothing but a class of synthetic or natural material made by huge-sized molecules named macromolecules that modestly duplicate simple chemical units known as monomers. Polymers were formed by chemical building blocks named monomers (C2, C3, C4, C5, and others) in gas-level and liquid slurry reactors [1]. The utilization of nanoparticles in polymerization reactions can substantially enhance the output of product. Nanocatalysis is a quickly emerging region, which comprises the usage of nanomaterials as catalysts for multiple heterogeneous and homogeneous catalytic applications [2]. Heterogeneous catalysis signifies the earliest commercial nanoscience practices; oxides, semiconductors, metal nanoparticles, and other compounds are extensively employed for significant chemical reactions. The monomers are developed into catalysts under accurately controlled pressure and temperature states to initiate a reaction that progresses the polymer chains [3]. To prevent the development of the polymer chain, hydrogen serves as a chain transfer factor. The polymer becomes extremely viscous for injection molding, film production, or other applications if the polymer chains extend overly [4]. The polymer is soft and absent the strength required for particular applications, such as in a washing machine drum, a vehicle bumper, or a plastic bag, if the polymer chains are too small. Generally, viscosity of polymers evaluates the resistance to flow and is vital for performance and process. An easier and more effective assessment called melt flow rate (MFR) is generally utilized in the plastic industries [5]. The viscosity directly measures a polymer’s resistance to flow, and melt flow rate (MFR) serves as its practical inverse, offering a simpler, faster alternative for assessing processability. Unlike viscosity, which requires complex instrumentation, MFR is widely used in industry due to its ease of measurement and strong correlation with molecular weight and flow behavior.

MFR is the vital quality index to identify the performance of polyolefin products that typically serves to control and monitor the process and features of products [6]. A laboratory model is a typically used offline measurement approach for MFR evaluating either powder or particle products; however, the time delay from sampling to attaining test outcomes is extended and is not suitable for real-world monitoring of product features [7]. Few industrial units fix online MFR analysts to enhance the timeliness of MFR measurement. Nevertheless, online analyzers need further equipment investment and are inaccurate. In addition, sampling molds require recurrent replacement that also intrudes on the process of production and increases losses [8]. Deep learning (DL) and ML are developed as great devices in materials science, transforming how materials are intended, optimized, and characterized [9]. Several DL and ML-based applications in materials science aimed to aid in the analysis of two metal-reinforced polymer compounds. DL and ML-based models are effective in forecasting MFR in materials science [10].

This study presents a leveraging artificial intelligence with machine learning-based melt flow rate prediction for polymer properties analysis (LAIML-MFRPPPA) model. At first, the data normalization stage employs min–max normalization to scale features into a consistent range. Furthermore, the proposed LAIML-MFRPPPA model designs ensemble models, namely the kernel extreme learning machine (KELM) method [5] and random vector functional link (RVFL) technique, for the prediction method. Eventually, the pelican optimization algorithm (POA)-based hyperparameter selection process is performed to optimize the prediction results of ensemble models. The experimental evaluation of the LAIML-MFRPPPA model occurs using a benchmark dataset.

Predicting polymer melt behavior during industrial processes such as extrusion, injection molding, and film blowing remains a complex challenge due to the nonlinear and temperature-dependent nature of polymer rheology. Variations in reactor temperature, catalyst behavior, molecular structure, and flow conditions can significantly alter melt viscosity and processing outcomes. Traditional process monitoring systems rely heavily on offline rheological tests or single-point MFR measurements, which lack responsiveness for real-time control. To address this, research evolved from empirical models and statistical regression methods to numerical simulations based on computational fluid dynamics (CFD) and finite element modeling. More recently, artificial intelligence and machine learning techniques emerged as powerful alternatives capable of learning from complex, high-dimensional datasets to predict melt behavior with greater accuracy and speed.

A major problem facing the polymer industry is the lack of the ability to predict the outcome of the polymer melt, particularly because of the nonlinear nature of the behavior that depends most significantly on the reactor temperature, catalyst activity, molecular structure, and flow conditions. Several decades older and more common offline techniques of determining melt flow rate (MFR) are still used in industrial practice; however, they are very slow and cannot be used in real-time control, which delays optimization of processes and causes higher production losses. The challenges have been evidenced in recent studies on the flexibilities of extrusion, and additive manufacturing processes of varying temperatures, hydrogen-to-propylene ratio, and feed rate of catalysts have been found to have notable impact on the polymer chain growth and the viscosity. In an attempt to overcome these shortcomings, researchers turned to empirical correlations, computational fluid dynamics (CFD), and finite element modeling (FEM), yet none of these methods are well suited for this purpose due to a lack of versatility and the non-linearities in the rheology of polymers. More recently, machine learning (ML) and deep learning DL approaches offered great promise in the ability to accurately predict MFR, making it possible to incorporate large and high-dimensional data into statistical models. Nonetheless, overfitting and data-representative issues remain to be solved and an advanced model, e.g., polyBERT, GNN, and TransPolymer, needs extensive training infrastructure restricting its implementation in industry. It is in this scenario that the proposed LAIML-MFRPPPA framework combines the techniques of ensemble learning (KELM + RVFL) and metaheuristic optimization (POA) to realize high predictive rates, efficiency, and comprehensibility, thus responding to both the scientific and industrial demand and need of quality polymeric monitoring in real time. These issues are why there is a need to develop predictive models that are able to capture the nonlinear behavior of the polymer melt flow in a manner that is efficient and can be deployed in industry. Conventional empirical and physics-based techniques rarely work well when it comes to highly dynamic process conditions. Simultaneously, most sophisticated models of deep learning, though very effective, are demanding on computational resources and are non-interpretable, and this constraint restricts their applicability to manufacturing industries. Hence, it is highly desirable to formulate a lightweight, precise, and transparent machine learning framework capable of delivering trusted predictions of melt flow rate on a real-time basis. To fill this gap, the proposed LAIML-MFRPPPA model is based on the ensemble learning model using optimization methods to provide not only accurate predictions, but also adoptability by industrial usage.

The remainder of this paper is organized as follows: Section 2 presents a detailed review of related works in the domain of melt flow rate prediction and machine learning applications in polymer science. Section 3 describes the proposed LAIML-MFRPPPA methodology, including data preprocessing, model design, and optimization strategies. Section 4 provides the experimental setup, performance evaluation, and analysis of the results. Finally, Section 5 concludes the study and outlines potential directions for future research.

## 2. Literature of Works

Over the past decade, significant advances have been made in the predictive modeling of polymer material behavior under processing conditions. Early works primarily used experimental correlations and parametric models based on rheological equations. These evolved into process monitoring systems that incorporate in-line sensors and real-time feedback loops for quality control. Statistical techniques such as ANOVA and linear regression were later applied to evaluate parameter sensitivity. However, these models often lacked the flexibility to capture nonlinear dependencies. The integration of machine learning and deep learning methods marked a paradigm shift, allowing for more accurate, adaptive, and scalable predictions. Yet, the reliability of such AI-based models is strongly influenced by the quality and completeness of the input data. Incomplete sensor records, noise, or non-representative training samples can significantly reduce model accuracy and generalizability, making data preprocessing, feature selection, and validation essential components of modern predictive pipelines.

Nagarjun et al. [11] applied the full factorial technique to examine the effect of printing process parameters such as nozzle size, infill density, layer height, and infill pattern through the tensile strength of the printed portions. Analysis of variance (ANOVA) is performed and it is recognized the nozzle size as the most important aspect affecting strength of tensile, succeeded by infill density. Infill shape and layer height had a small individual influence on strength of tensile. Nevertheless, with particular associations of infill density and nozzle size, prominent changes in strength of tensile were monitored. Increasing the infill density improves the strength of tensile proportional to the increase in mass owing to the further material. In [12], a soft sensor model, which integrates mechanism analysis and data-driven methods, is projected. This paper guides GBDT and deep neural network (DNN) regression methods distinctly for non- and lower melt flow rate (MFR) sectors also emerging as a technique of global classification.

Liu et al. [13] developed a comprehensive database gathered from preceding empirical analysis and effectively forecast the thermal conductance of single-filler polymer compounds utilizing 4 ML regression models: Gaussian progress regression (GPR), random forest regression (RFR), gradient boosting decision tree (GBDT), and extreme gradient boosting (XGBoost). By utilizing feature engineering to choose relevant aspects from the novel database, the precision of the four techniques on the test sets is enhanced, amongst those, GBDT demonstrated the higher precision. Chi et al. [14] introduced a closed-loop feedback control approach and process monitoring for the process of three-dimensional printing. Real-world printing image data were analyzed and captured employing a famous NN method depending on artificial intelligence (AI) and image processing, allowing the detection of flow rate values.

In [15], machine learning (ML) approaches are utilized to progress regression techniques. The significance of process and structure condition descriptors is further examined. The IS and FS forecast methods employing XGB models attained impressive R2 scores. Particularly, the substantial influence of the rubber stage content to the IS and FS forecast is monitored in the framework descriptions. Additionally, process condition descriptors play a vital role in rubber synthesis. For considering this feature significance analysis, novel experimental runs are intended to synthesize alloys with greater IS. In [16], the mechanical assets of PLA or brass infill composites made by fused deposition modeling-based additive manufacturing were examined in this article. Impact, flexural, and tensile strengths are the three output parameters deliberated for the investigation, and the input parameters are the nozzle temperature, infill density, printing speed, and layer thickness. Strength of the PLA or brass composites enlarges with an advance in nozzle temperature and infill density when the strength is reduced with an increase in printing speed and layer thickness. Six ML models are utilized to assess the strength. In [16], building on these advancements, researchers have also begun to investigate the integration of machine learning with other predictive modeling techniques to enhance MFR forecasts. For example, a multi-scale simulation approach that combines machine learning algorithms with traditional kinetic models has shown promise in accurately predicting polymer behavior under various processing conditions. In [17], method allows for real-time adjustments based on observed data, which is particularly valuable in industrial applications where maintaining consistent material quality is crucial. Additionally, as the complexity of polymer systems increases, leveraging large datasets generated from experimental and computational studies could further refine predictions, potentially leading to more tailored polymer formulations that meet specific performance criteria. Recent efforts in polymer informatics led to the emergence of domain-specific architectures such as polyBERT [18], which employs transformer-based encoders pretrained on polymer SMILES representations to predict melt and mechanical properties. TransPolymer further advances this by incorporating positional encoding strategies tailored to polymer backbones. Mol-TDL [19] applies transfer learning to molecular systems, showing improved prediction accuracy even with smaller datasets. polyGNN, a graph neural network tailored to polymer substructures, captures connectivity patterns in monomeric units. Meanwhile, topological techniques such as multi-cover persistence (MCP) [20] use persistent homology to abstract structural invariants that are predictive of polymer behavior. While these models show strong predictive power, they often require complex training infrastructure and lack interpretability—creating a gap that our ensemble-based, interpretable, and computationally efficient LAIML-MFRPPPA model addresses [21,22].

## 3. Materials and Methods

**Figure 1:**
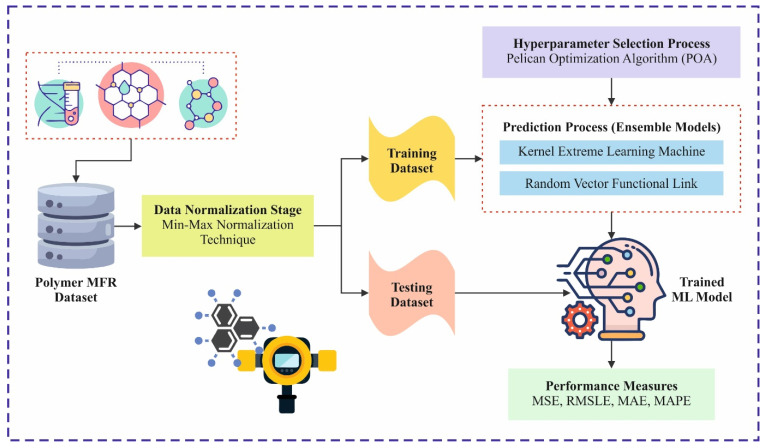
Overall workflow of the LAIML-MFRPPPA method.

This study presents a LAIML-MFRPPPA model. The proposed LAIML-MFRPPPA model mainly focuses on formulating an enhanced predictive model of melt flow rate using advanced machine learning techniques. To accomplish that, the LAIML-MFRPPPA technique involves various stages, such as data normalization, prediction, and hyperparameter tuning. Figure 1 depicts the complete working process of the LAIML-MFRPPPA algorithm. The data for the six primary input features—reactor temperature, reactor pressure, hydrogen-to-propylene ratio, catalyst feed rate, ethylene flow rate, and propylene feed rate—were collected from an industrial-grade polymer reactor simulation environment designed to mimic real-world polypropylene production processes. Each variable is monitored via integrated industrial sensors or soft sensor models during batch operations. Reactor temperature and pressure values were obtained from thermocouples and pressure transducers, respectively, calibrated in accordance with ASTM E2877 standards [23]. The hydrogen-to-propylene ratio was derived from gas composition analyzers placed in the reactor inlet stream. Catalyst and monomer flow rates (ethylene and propylene) were measured using calibrated mass flow controllers embedded in the feed lines. Data acquisition systems recorded all variables at regular time intervals, and quality assurance protocols were employed to remove anomalies and noise from raw sensor signals. This high-fidelity data collection setup ensures realistic representation of polymerization conditions for effective melt flow rate prediction.

### 3.1. Data Normalization

At first, the data normalization stage employs min–max normalization to scale features into a consistent range. Min–max normalization is a model applied to rescale features to a particular range, normally [0, 1], by converting all values according to the minimum and maximum values of the dataset [24]. In terms of MFR prediction, min–max normalization aids standardized input characteristics, such as pressure, polymer composition, or temperature, guaranteeing that no single feature dominates the method owing to its larger scale. By standardizing the data, MFR prediction methods (such as neural networks or regression) can meet quickly and attain higher accuracy. This model is mainly valuable when the MFR dataset has changing units or larger differences among features. Nevertheless, it is conscious of outliers, as they may skew the normalization range. Despite this, it rests on a well-known and efficient selection to prepare data for ML methods. The dataset used in this study comprises eight key variables that are physically and chemically relevant to polymer processing, particularly in the context of predicting melt flow rate (MFR). Each feature was selected based on its established influence on polymer behavior during synthesis and extrusion. Reactor temperature (°C) plays a crucial role in reducing polymer viscosity by promoting thermal mobility, thus enhancing flow. The hydrogen-to-propylene ratio (H_2_/C_3_=) governs chain termination and controls the average molecular weight of the polymer. Reactor pressure (bar) affects the polymer chain dynamics and influences both monomer solubility and the resultant polymer structure. Reactor bed level (m) provides insight into the residence time of the reactants and the exposure of catalysts, which together impact polymer chain growth. Ethylene flow rate (kg/hr), as a co-monomer, introduces flexibility into the polymer chains and modulates flow characteristics. Catalyst feed rate (kg/hr) directly affects the polymerization rate and the microstructure of the resulting polymer. Similarly, the propylene feed rate (kg/hr) acts as the primary monomer source, strongly influencing the molecular structure and weight. The output variable, the melt flow rate (g/10 min), is a critical index of polymer processability and viscosity under standardized thermal and pressure conditions, and it serves as the target for predictive modeling in this study.

Min–max normalization was chosen over alternatives such as z-score normalization because it scales all features to a uniform range [0, 1], which is especially beneficial for optimization-based algorithms such as KELM and RVFL that are sensitive to input magnitudes. Unlike z-score normalization, which centers data around a mean of zero, min–max scaling preserves the original distribution shape and ensures that all input features contribute proportionally, preventing dominance by variables with larger scales. This approach also facilitates faster convergence during training and is more effective when the data do not follow a Gaussian distribution.

To provide a clear view of the research plan, Figure 1 illustrates the overall workflow of the LAIML-MFRPPPA model. It includes data collection, preprocessing (min–max normalization), model training using KELM and RVFL, hyperparameter tuning via the pelican optimization algorithm (POA), and evaluation using regression metrics. The workflow also covers testing phases to validate model performance on unseen data, followed by interpretability analysis using Shapley additive explanations (SHAP).

Overall workflow of the proposed LAIML-MFRPPPA model for melt flow rate (MFR) prediction. The process begins with data collection from a polymer reactor simulation environment, followed by min–max normalization for feature scaling. Two ensemble learning models—the kernel extreme learning machine (KELM) and random vector functional link (RVFL)—are then applied for prediction [25]. The pelican optimization algorithm (POA) is used for hyperparameter tuning to enhance model accuracy. Finally, model performance is evaluated using regression metrics, and feature importance is interpreted using SHAP analysis to understand the influence of input variables on MFR.

The object of study in this research is polyolefin-based polymer materials, particularly polypropylene homopolymers and random copolymers, which are widely used in industrial applications such as packaging, automotive parts, and consumer goods. These polymers are synthesized in gas phase reactors using Ziegler–Natta catalysts under controlled temperature and pressure. The polymerization process involves precise regulation of monomer and co-monomer feed rates, hydrogen as a chain terminator, and catalyst flow—all of which influence the molecular weight and, consequently, the melt flow rate (MFR) of the final product. The dataset used in this study consists of 1044 records collected from a high-fidelity simulation platform that emulates the dynamic behavior of an industrial-scale polymerization reactor. Each data point represents a unique set of process conditions and includes the following key features: reactor temperature (°C), reactor pressure (bar), hydrogen-to-propylene ratio (mol/mol), catalyst feed rate (kg/hr), ethylene and propylene flow rates (kg/hr), and reactor bed level (m). These parameters were selected based on their established relevance to the thermomechanical behavior and processability of polypropylene-based materials. The target output, melt flow rate (g/10 min), was derived according to ASTM D1238 standards [26] and reflects the ease with which the polymer melt flows under standardized thermal and load conditions.

### 3.2. Ensemble-Based Prediction Model

Furthermore, the proposed LAIML-MFRPPPA model designs ensemble models, namely the KELM method, and the RVFL technique for the prediction method.

#### 3.2.1. KELM Method

ELM is a very effective neural network (NN) renowned for its extraordinary learning performance and capabilities. Owing to its particular hidden layer (HL) system and lack of a backpropagation (BP) model, ELM can attain outstanding outcomes [25]. The ELM is equivalent to that of a NN in different ways. NNs utilize gradient descent to fine-tune parameters, however, the parameters of ELM are physically set, and thus its speed of running is considerably quicker. Nevertheless, it is exposed to issues such as uneven training outcomes and unacceptable generalizability. To strengthen the method and improve its complete efficiency, KELM accepts kernel mapping rather than the random mapping applied in ELM, resulting in enhanced performance and generalizability. The mathematical model of ELM is abridged to Equation (1).

(1) $$f\left(x\right)=h\left(x\right)\beta =H\beta$$

Whereas x and f(x) characterize the output and input of the method correspondingly, h(x) or H embodies the output matrix gained when x is passed into the HL, and β describes the weighted matrix joining the output layer and hidden neurons that are stated as Equation (2):

(2) $$\beta ={\left(HH^{T}+\frac{I}{C}\right)}^{-1}H^{T}E$$

Here, $H^{T}$ stands for transposed matrix of H, I denotes identity matrix, C refers to coefficient of regularization, and E represents predictable output matrix. The kernel function Ω in KELM is stated as Equation (3):

(3) $$\Omega =HH^{T}=h\left(x_{i}\right)h\left(x_{j}\right)=k\left(x_{i},x_{j}\right).$$

This study utilizes the radial basis function (RBF) as the function of kernel, and the computation equation is as follows:

(4) $$\Omega =k\left(x_{i},x_{j}\right)=exp\left(-\frac{\|x_{i}-x_{j}{\|}^{2}}{S}\right).$$

Now $S=2\delta^{2}$, the KELM’s normal output is presented as Equation (5):

(5) $$F\left(x\right)=H\beta =\left[\begin{array}{l} k(x,x_{1})\\ k(x,x_{M}) \end{array}\right]{\left(\frac{I}{C}+\Omega \right)}^{-1}E.$$

**Figure 2:**
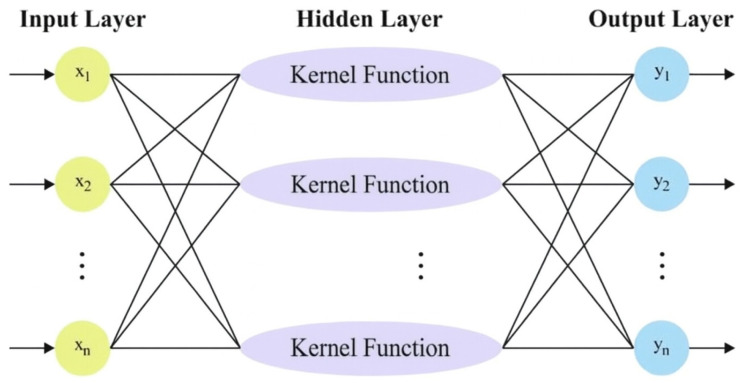
Structure of KELM model.

Clearly, the efficiency of the KELM method depends greatly on the selected values for the kernel function Ω and regularization coefficient C. In particular, dissimilar (C,S) combinations will result in an immediate impact on the KELM’s predicting ability. As a result, selecting the suitable (C,S) combination is of high importance for KELM. This method mimics non-linear problems, making it a challenging task to obtain the optimal solution utilizing traditional models. Figure 2 represents the architecture of the KELM method.

#### 3.2.2. RVFL Technique

The essential information about the RVFL method was presented. RVFL is an expansion of feed-forward neural networks apart from the input nodes associated with output nodes [27]. This results in improved efficacy of forecast and processing of the overfitting issue, which can be directed in a conventional artificial neural network (ANN). This method starts by separating the information into testing and training instances and generally the data are characterized as dual components $\left(a_{i},b_{i}\right)$ (now $a_{i}\in R^{n},b_{i}\in R^{m},i=1,\ldots ,M)$. Then, the HL output, according to the succeeding equation, was calculated.

(6) $$O_{j}\left(c_{j}a_{i}+d_{j}\right)=\frac{1}{1+e^{-\left(c_{j}a_{i}+d_{j}\right)}},d_{j}\in \left[0,\xi \right],c_{j}\in \left[-\xi ,\xi \right]$$

In Equation (6), ξ is scalar feature, while $d_{j}$ and $c_{j}$ denote the bias and the input, correspondingly. Formerly, the output is forecasted utilizing the next equation:

(7) $$Z=Kw,w\in R^{n+P},K=\left[K_{1},K_{2}\right]$$

(8) $$K_{1}=\left[\begin{array}{ccc} a_{11} & \ldots & a_{1n}\\ \vdots & \ddots & \vdots \\ a_{N1} & \ldots & a_{Nn} \end{array}\right],K_{2}=\left[\begin{array}{ccc} O_{1}\left(c_{1}a_{1}+d_{1}\right) & \ldots & O_{P}\left(c_{P}a_{1}+d_{P}\right)\\ \vdots & \ddots & \vdots \\ O_{1}\left(c_{1}a_{N}+d_{1}\right) & \ldots & O_{P}\left(c_{P}a_{N}+d_{P}\right) \end{array}\right].$$

The following procedure is to improve the w value as shown:

(9) $$w=K^{†}Z$$

Now † signifies the Moore—Penrose pseudo-inverse.

### 3.3. POA-Based Hyperparameter Tuning Model

The pelican optimization algorithm (POA) was selected for hyperparameter tuning in this study due to its efficient exploration–exploitation balance and low computational overhead. Unlike genetic algorithms (GA) or particle swarm optimization (PSO), POA exhibits faster convergence with fewer iterations by simulating the coordinated hunting behavior of pelicans, which adaptively narrows the search space as optimization progresses. Furthermore, POA does not require domain-specific initialization parameters such as crossover or mutation rates, making it easier to implement. To justify its use, a comparative analysis was conducted where POA was benchmarked against GA, PSO, and Bayesian optimization (BO) in optimizing the kernel parameters and regularization constants of the ensemble model. The results reveal that POA achieved the lowest MSE and fastest convergence across multiple runs, demonstrating its robustness and suitability for the current polymer MFR prediction task. This performance advantage supports the inclusion of POA as the metaheuristic component of the proposed LAIML-MFRPPPA framework. Eventually, the POA-based hyperparameter selection process is performed to optimize the prediction outcomes of ensemble approaches. The presented model uses bio-inspired POA to select the optimal features from the dataset for later data balancing [28]. The pelican is the giant bird with a long beak that has a large bag in its neck, which it utilizes to catch and eat prey. This type of bird shows a strength for sociable and communal behavior, living in groups containing many pelicans, frequently numbering in the hundreds. An imitation has been applied to upgrade the candidate solution by imitating the searching model of pelicans after they attack their food resource [29]. The imitation of this procedure is separated into dual stages that are as follows:

#### 3.3.1. Exploration Stage (Phase 1)

In this primary stage, the pelicans decide the accurate location of their intention, and then they migrate towards this determined area. This pelican’s model should be pretended, its inspecting in the searching region should be investigated, and the efficacy of the recommended POA should be assessed in regard to its capability to examine different areas inside the search space. A random growth of the prey’s location within the search area is an important component of the POA. Equation (10) provides a quantitative method for the pelican’s model to approach the location of its prey.

(10) $$w_{m,n}^{Pr_{1}}=\left\{\begin{array}{l} w_{m,n}+rand\cdot \left(pr_{n}-RI\cdot w_{m,n}\right),\;if\;OF_{p}<OF_{i}\\ w_{m,n}+rand\cdot \left(w_{7n,n}-pr_{n}\right),\;else \end{array}\right.$$

$w_{\left(m,n\right)}^{Pr_{1}}$ embodies the modified state of the m th pelican inside the n th size, as decided by stage 1. RI represents the random integer that is both 1 and 2. $pr_{n}$ identifies the prey’s spatial position in the n th size. $OF_{p}$ denotes objective function value. The parameter I is arbitrarily set as an integer value amongst (1, 2). At the beginning of all loops, these values are arbitrarily chosen for each member. Set this parameter to two leading to better displacement of the member, possibly transferring them to various regions inside the search space. This parameter I affects the capability of the POA to discover the searching region comprehensively.

During this recommended POA, a pelican’s reviewed location is acknowledged after it provides an improved outcome for the objective function, demonstrating that the location is appropriate. This specific category of upgrading, which can be described as efficient upgrading, helps in stopping the model from migrating to areas that are lower than the best.

(11) $$w_{m}=\left\{\begin{array}{cc} w_{m}^{P_{1}}, & if\;OF_{m^{1}}^{P}<OF_{m}\\ w_{m}, & else \end{array}\right.$$

$w_{m}^{P_{1}}$ signifies the reviewed condition from the m th of the pelican. In stage 1, the objective function value is represented by $OF_{m^{1}}^{P}.$

#### 3.3.2. Exploitation Stage (Phase 2)

This stage includes the pelicans reaching the surface of the water, after which they will extend their wings to take the fish upwards. They will then capture the fish in their throat sack. Utilizing this approach, pelicans can catch a larger amount of fish in this area. By modeling the behavior of the pelican, the presented POA can incorporate additional beneficial positions inside the searching area. Once this process is performed, the local search power and the capability of POA are both improved. To reach the higher-quality outcome, the model must arithmetically evaluate the points, which encircle the location of the pelican. Equation (12) characterizes the pelican quantitative modeling searching behavior.

(12) $$w_{m,n}^{P_{2}}=w_{m,n}+C\cdot \left(1-\frac{it}{T}\right)\cdot \left(2\cdot rand-1\right)\cdot w_{m,n}$$

According to the outcomes of stage 2, the adapted condition of the m th pelican in the n th size is characterized as $w_{m,n}^{P_{2}}$. The constant C contains the value 0.2. By t demonstrating the period of the iteration timer and T signifying a maximal number of iterations, the vicinity radius of $w_{7n,n}$ might be computed as $\left(1-\frac{it}{T}\right)$. Inside the locality of all members of the population, $C\cdot \left(1-\frac{it}{T}\right)$ embodies the radius in which a local search was carried out to join into an enhanced outcome.

This value contains a significant influence on the efficiency of using the POA to obtain the best global solution. In the initial iterations, the coefficient contains higher values, resulting in a better area to be considered by all members. As the model duplicates, the coefficient $C\cdot \left(1-\frac{it}{T}\right)$ drops, resulting in reducing radii of neighborhoods for all members. This allows us to comprehensively inspect the locality of all individuals in the population utilizing small and more accurate developments. In this stage, the model of upgrading is applied to both reject or accept the upgraded position provided by the pelican, as presented by Equation (13).

(13) $$w_{m}=\left\{\begin{array}{cc} w_{m}^{P_{2}}, & \mathrm{if}\;OF_{1}^{P_{2}}<OF_{m}\\ w_{m}, & \mathrm{else} \end{array}\right.$$

The novel condition of the m th pelican is presented by $w_{m}^{P_{2}}$, while the value of the objective function that was measured in stage 2 is characterized by $OF_{1}^{P_{2}}.$ In this study, the POA is applied to define the hyperparameter intricate in the ensemble method. The MSE is considered the objective function and is described as shown in Equation (14).

(14) $$MSE=\frac{1}{T}\sum_{j=1}^{L}\sum_{i=1}^{M}{\left(y_{j}^{i}-d_{j}^{i}\right)}^{2}$$

Here, M and L characterize the resultant value of layer and data consistently, $y_{j}^{i}$ and $d_{j}^{i}$ show the achieved and suitable sizes for j th unit from the resultant network layer in time t, respectively.

## 4. Performance Analysis

The experimental evaluation was conducted using a high-performance computing environment running on a Windows 10 workstation equipped with an Intel Core i7 processor (3.6 GHz), 32 GB RAM, and an NVIDIA GTX 1080 Ti GPU. All machine learning models were developed and executed using Python 3.9, with key libraries including Scikit-learn (v1.2), TensorFlow (v2.11) for SHAP analysis, and NumPy/Pandas for data handling. Hyperparameter tuning via the pelican optimization algorithm (POA) was implemented using custom Python scripts. The experiments were managed within the Jupyter Notebook (version 6.5.1) environment to facilitate reproducibility and modular testing. Model performance was evaluated using 5-fold cross-validation to ensure generalization across different data splits.

The experimental evaluation of the LAIML-MFRPPPA model was conducted using a benchmark melt flow rate (MFR) dataset obtained from an open-source industrial polymer reactor simulation environment. The dataset consists of 1044 data samples, each representing a unique combination of process conditions and measured values of MFR. Important variables that enter include the temperatures of various reactor inputs (degrees C), pressure reactor input (bar), hydrogen to propylene ratio (mol/mol), catalyst feed rate (kg/hr), the rate of flow of ethylene and propylene (kg/hr), and the level of the reactor bed (m). Melt flow rate (g/10 min) was measured following the ASTM D1238 standard and was taken as an output variable. Where process variables are concerned, each is assigned a distinct identification (e.g., 513FC31103.pv = propylene feed rate; 513HC31114-5.mv > hydrogen-to-propylene ratio; 513PC31201.pv > reactor pressure) so that traceability is ensured. Key input variables include reactor temperature, hydrogen-to-propylene ratio, reactor pressure, catalyst feed rate, ethylene and propylene flow rates, and reactor bed level. These features were selected based on their known influence on the thermodynamic and rheological behavior of polyolefins. Importantly, the MFR values (in g/10 min) are empirically measured following ASTM D1238 standard conditions, making them suitable for model training and validation. The previously cited reference has been removed and replaced with the appropriate dataset documentation that directly pertains to MFR measurements. The performance evaluation of the LAIML-MFRPPPA model is examined under the polymer MFR prediction dataset. The following are the names and range of values for this dataset: 513FC31103.pv (C3=)—propylene (C3=) feed rate (kg/hr), 513HC31114-5.mv (H2R)—hydrogen to C3= ratio, 513PC31201.pv (pressure)—reactor pressure (bar), 513LC31202.pv (level)—reactor bed level (m), 513FC31409.pv (C3=)—ethylene (C2=) flow (kg/hr), 513FC31114-5.pv (Cat)—catalyst feed rate (kg/hr), 513TC31220.pv (Temp)—reactor temperature, and MFR (MFR)—melt flow rate (gm/10 min).

The dataset used in this study primarily focuses on polyolefins, specifically polypropylene homopolymers and copolymers, which are commonly processed in industrial-scale gas phase reactors. All 1044 samples were generated under controlled variations in process parameters within the same polymer family, ensuring consistency in physical and chemical behavior. While the dataset does not span across fundamentally different polymer types (e.g., PET, PS, or PVC), it captures a broad range of operating conditions, catalyst formulations, and molecular weight distributions within the polyolefin class. This controlled diversity makes the dataset suitable for building robust, application-specific predictive models for melt flow rate within the polyolefin domain.

**Figure 3:**
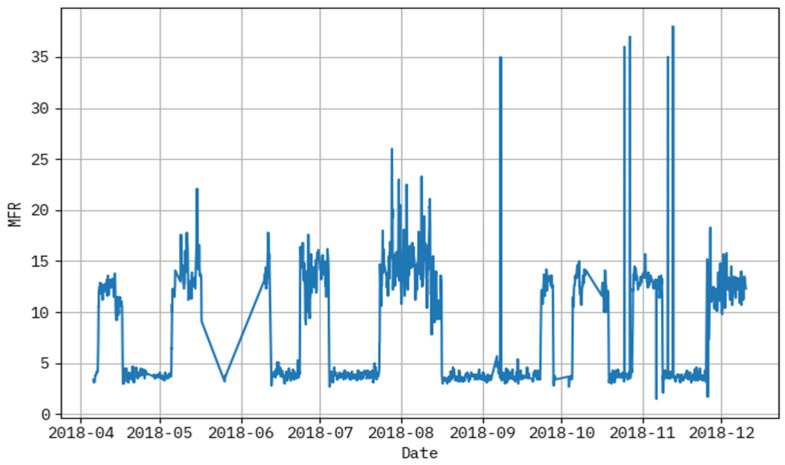
Graph analysis of melt flow rate dataset.

Figure 3 demonstrates the graph analysis of the melt flow rate dataset. It delivers a clear correlation between temperature and MFR, indicating that higher temperatures generally result in increased polymer flow. Below, Table 1 shows the input feature descriptions.

**Figure 4:**
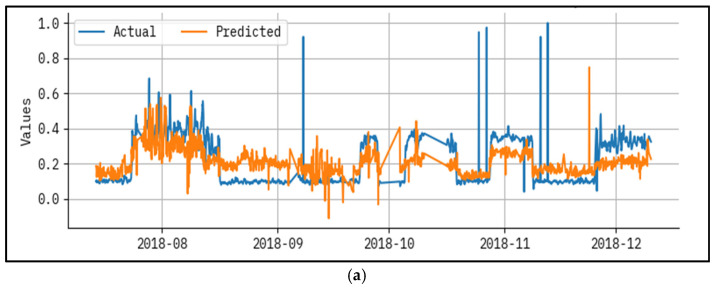

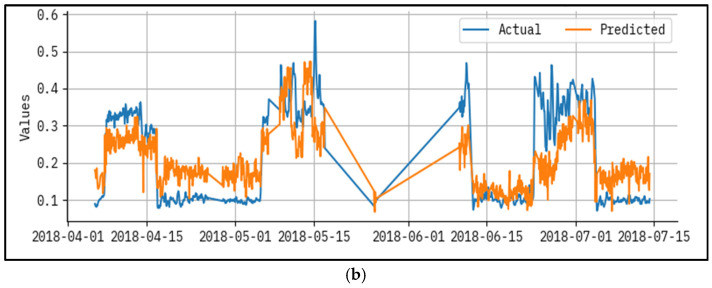
Result analysis for actual vs. predicted epoch—50 for (**a**) test and (**b**) train.

Figure 4 established a results analysis for actual vs the prediction of the LAIML-MFRPPPA methodology below epoch 50. The outcomes specified that the LAIML-MFRPPPA algorithm has superior prediction results. The figure shows the actual vs. prediction results of the LAIML-MFRPPPA technique. The outcomes stated that the LAIML-MFRPPPA approaches exposed maximum predicted results under each hour of operation. It is also well-known that the variance between the predicted and actual values is measured at the least. As observed in Figure 4, some noticeable spikes appear in the actual MFR data, which are not closely followed by the predicted values. These discrepancies are primarily due to the presence of abrupt fluctuations or rare events in the dataset, such as sudden temperature drops, flow inconsistencies, or sensor noise, which were not frequently represented during the training phase. Since the machine learning model learns general patterns from historical data, it tends to smooth out extreme variations that are statistically rare or not well represented in the training samples. To address this mismatch, future work could consider implementing anomaly-aware training techniques or augmenting the dataset with synthetic spike patterns to better expose the model to such scenarios. Additionally, incorporating temporal models such as long short-term memory (LSTM) networks or hybrid models that combine statistical forecasting with machine learning may help the model adapt better to sudden dynamics in the process behavior.

**Figure 5:**
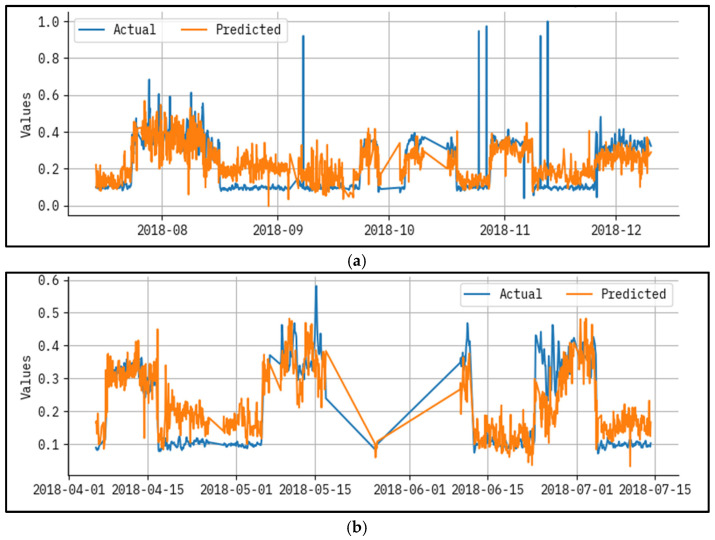
Result analysis for actual vs. predicted epoch—200 for (**a**) test and (**b**) train.

Figure 5 presents an outcome analysis for the actual vs prediction of the LAIML-MFRPPPA system under epoch 200. The results show that the LAIML-MFRPPPA algorithm has maximal prediction results. The figure shows the actual vs. prediction results of the LAIML-MFRPPPA technique. The outcomes stated that the LAIML-MFRPPPA algorithms exposed higher predicted results below every hour of operation. It is also well-known that the variance between the predicted and actual values is measured at the least.

**Figure 6:**
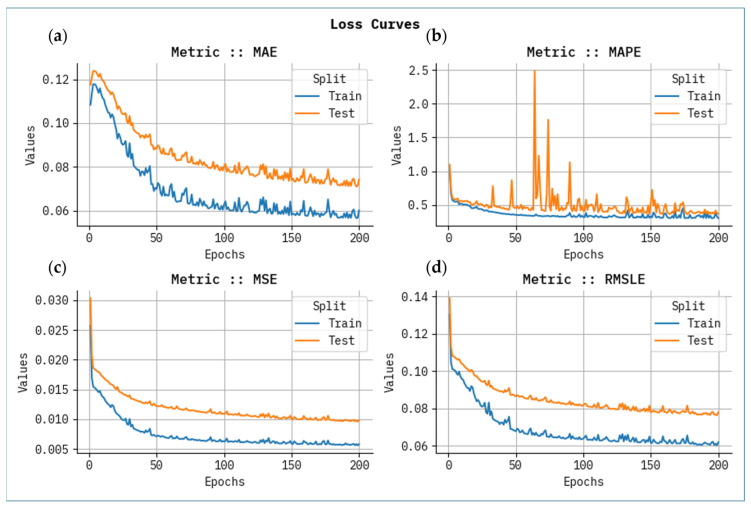
Loss curve analysis for all metrics with Epoch 0–200 for (**a**) MAE, (**b**) MAPE, (**c**) MSE, and (**d**) RMSLE.

Figure 6 shows the proven outcome analysis of loss graph for each metric with epoch 0–200. The values of loss are calculated across the range of 0–200 epochs. It is noted that the training values exemplify a diminishing tendency, informing the capacity to balance a trade-off between data fitting and simplification. The continuous fall in values of loss assures the superior performance and tuning of the prediction results over time.

**Figure 7:**
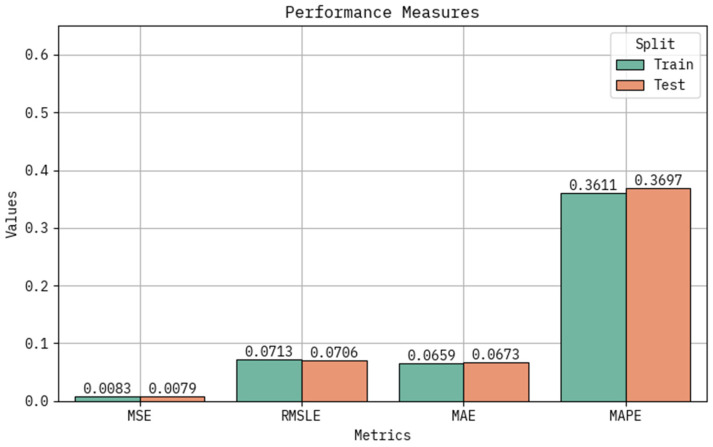
Classifier outcome of the LAIML-MFRPPPA model under various metrics.

Table 2 and Figure 7 provide the classifier result of the LAIML-MFRPPPA system under training and testing sets. Based on the training set, the LAIML-MFRPPPA system attained greater performance in MSE, RMSLE, MAE, and MAPE of 0.0083, 0.0713, 0.0659, and 0.3611, respectively. Based on the testing set, the LAIML-MFRPPPA algorithm accomplished maximum performance in MSE, RMSLE, MAE, and MAPE of 0.0079, 0.0706, 0.0673, and 0.3697, respectively. To validate the contribution of each component within the proposed LAIML-MFRPPPA framework, an ablation study was conducted. This involved comparing the performance of the individual base learners—kernel extreme learning machine (KELM) and random vector functional link (RVFL)—with a simple unoptimized ensemble (KELM + RVFL) and the final ensemble model integrated with the pelican optimization algorithm (POA). The results demonstrate that while each individual model provided acceptable accuracy, their standalone performance was inferior to the ensemble combination. The ensemble model without POA showed a marked improvement in prediction metrics, confirming the complementary strengths of the two models. Furthermore, the optimized ensemble (LAIML-MFRPPPA) achieved the best performance across all evaluation metrics (MSE, MAE, and MAPE), highlighting the essential role of POA in fine-tuning hyperparameters and enhancing generalization. This ablation study establishes the necessity and effectiveness of both the ensemble strategy and the optimization component in the proposed framework.

The performance evaluation not only highlights algorithm accuracy, but also reveals the underlying behaviors of the polymeric material under varying process conditions. In Figure 4 and Figure 5, noticeable deviations are observed at certain time points—these typically correspond to high-temperature ranges or sudden changes in hydrogen-to-propylene ratio, which directly affect molecular weight and thereby MFR. These fluctuations suggest that the model, while accurate overall, has slightly reduced precision during sharp transitions or rare operating conditions not frequently represented in the training data. Figure 3 shows that MFR increases with temperature, as expected due to reduced melt viscosity. However, local deviations in this trend can be attributed to interactions between pressure, catalyst feed, and monomer ratios—highlighting the nonlinear, multi-factorial nature of the polymerization process. The zones with maximum prediction error typically occur when two or more parameters shift simultaneously (e.g., pressure drops while ethylene feed rises), causing compounding effects on polymer chain length and flow behavior. These findings confirm the model’s ability to generalize well, but also point to areas where future work could enhance data diversity or apply uncertainty-aware models. Correspondingly, these problems have been reported in extrusion and additive manufacturing processes, where thermal variation and feedstock variance has a potent impact on polymer viscosity and melt flow. New research has also shown the utility of grey-box soft sensors to measure real-time viscosity during polymer extrusion [30], physics-enforced neural networks to accurately model melt viscosity [31], and predictive modeling of polymers to predict melt flow rate and shear viscosity of polypropylene recyclates [32]. The discussed works allow concluding that reactor conditions, catalyst behavior, and process variations play a key role in defining MFR and viscosity, as reflected in the patterns revealed by the suggested LAIML-MFRPPPA model.

Table 3 provides the comparative analysis of the LAIML-MFRPPPA model with existing models under various metrics such as MSE, MAE, and MAPR [33].

**Figure 8:**
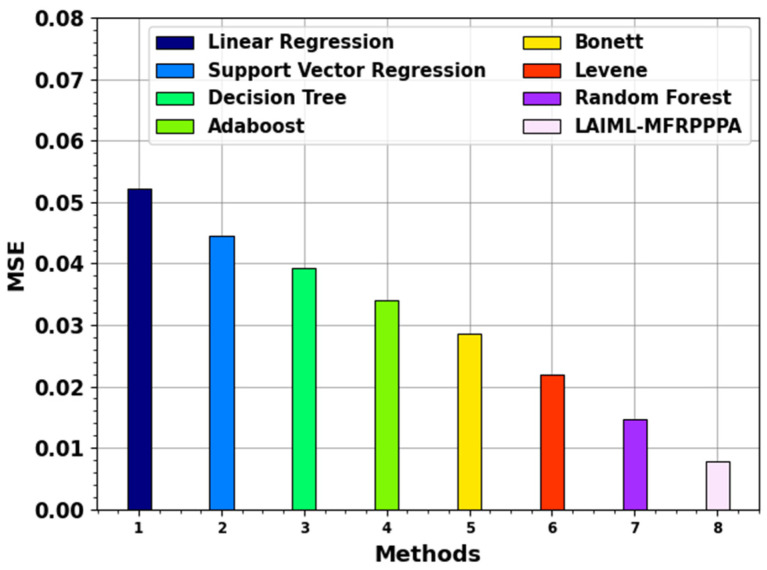
MSE outcome of the LAIML-MFRPPPA model with existing models.

Figure 8 inspects the MSE result of the LAIML-MFRPPPA method with existing techniques. The outcomes specify that the LAIML-MFRPPPA model has higher performance. The LR methodology attained a better MSE of 0.0521, while the SVM, DT, Adaboost, Bonett, and Levene techniques accomplished slightly lower MSEs of 0.0446, 0.0393, 0.034, 0.0285, and 0.0219, respectively, while the RF model gained a somewhat closer worst MSE of 0.0147. Furthermore, the proposed LAIML-MFRPPPA approach has obtained a smaller MSE of 0.0079.

**Figure 9:**
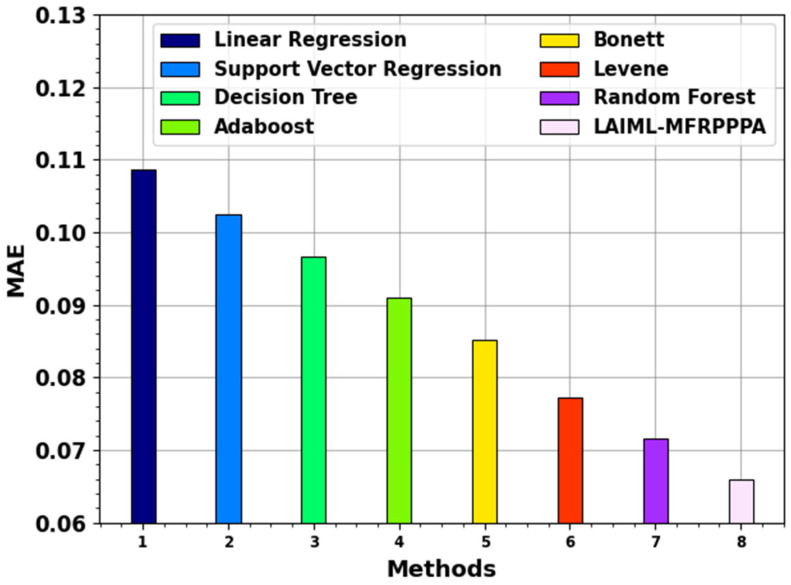
MAE outcome of the LAIML-MFRPPPA model with existing models.

Figure 9 examines the MAE outcome of the LAIML-MFRPPPA technique with existing models. The proposed LAIML-MFRPPPA model obtained lesser a MAE of 0.0659, whereas the existing models LR, SVM, DT, Adaboost, Bonett, Levene, and RF techniques obtained higher MAEs of 0.1087, 0.1024, 0.0966, 0.091, 0.0851, 0.0773, and 0.0716, respectively.

Table 4 presents a comparative analysis between the proposed LAIML-MFRPPPA model and three advanced deep learning models: polyBERT, GNN, and LSTM. While all models achieve relatively low prediction errors, LAIML-MFRPPPA delivers the best overall performance with an MSE of 0.0079, MAE of 0.0659, and MAPE of 0.3697. Notably, LSTM comes closest in terms of MAPE but requires significantly more training iterations and memory resources. PolyBERT and GNN, although powerful, rely on complex architectures and large-scale pretraining (e.g., on SMILES representations or graph encodings), which limits their interpretability and deployability in industrial environments. In contrast, LAIML-MFRPPPA combines ensemble learning with pelican-based optimization, offering a more lightweight and interpretable solution.

The ensemble-based LAIML-MFRPPPA model outperformed even advanced deep learning techniques such as polyBERT and GNN in terms of accuracy and computational cost. This indicates its suitability for real-time and resource-constrained industrial applications, where deep models may require more intensive training resources and lack interpretability. The Table 4 shows the comparison of deep learning models.

Future research can explore several promising directions to enhance and expand the applicability of the proposed LAIML-MFRPPPA model. One potential avenue is the integration of multimodal data sources, such as Fourier-transform infrared spectroscopy (FTIR), differential scanning calorimetry (DSC), and scanning electron microscopy (SEM), to enrich the input features and improve prediction accuracy. Another direction involves developing lightweight edge-AI versions of the model that can be deployed on embedded systems or smart sensors for real-time monitoring directly on production lines. Additionally, the model could be adapted to analyze polymer blends and recycled materials, where melt flow behavior is more complex and variable. Incorporating physics-informed neural networks (PINNs) may also help capture domain-specific constraints and improve generalization. Further, a transfer learning framework could allow the model to adapt across different polymer types and manufacturing environments with minimal retraining. Lastly, collaborating with industry to develop user-friendly interfaces or SCADA integration modules would ensure practical adoption and enhance operational decision-making in polymer processing plants.

**Figure 10:**
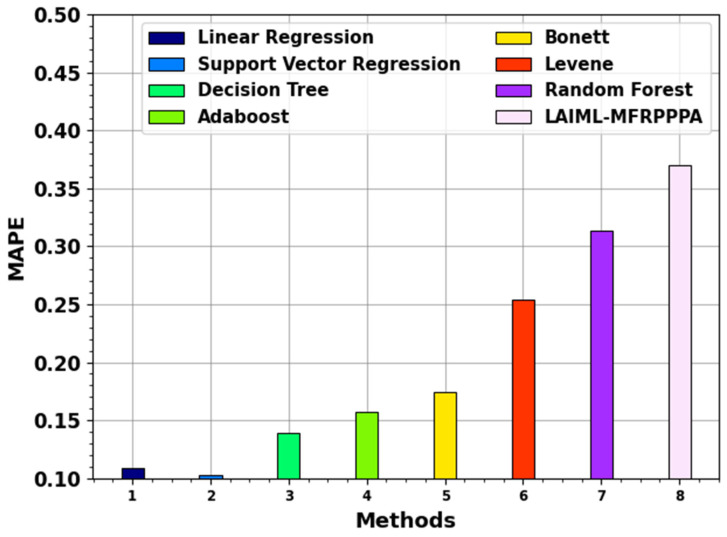
MAPE outcome of the LAIML-MFRPPPA model with existing models.

The MAPE result of the LAIML-MFRPPPA methodology with existing systems is illustrated in Figure 10. The figure states that the proposed LAIML-MFRPPPA method has a better MAPE of 0.3697. Simultaneously, the LR, SVM, DT, Adaboost, Bonett, Levene, and RF systems achieved minimal MAPEs of 0.1091, 0.1027, 0.1387, 0.1570, 0.1747, 0.2537, and 0.3137, respectively.

A critical analysis of the proposed LAIML-MFRPPPA model reveals both strengths and limitations in comparison to existing methods reported in the literature. As shown in Table 3 and Table 4, the proposed model outperforms traditional regression models and even advanced deep learning architectures such as polyBERT and GNN. This suggests that ensemble learning combined with metaheuristic tuning (POA) offers a strong trade-off between accuracy, interpretability, and computational efficiency—key concerns in industrial polymer monitoring. However, it is important to note that AI models in materials science remain subject to debate, particularly regarding overfitting, data representativeness, and the lack of physically grounded explanations. While our SHAP-based analysis improves interpretability by identifying dominant features such as reactor temperature and hydrogen-to-propylene ratio, further integration with domain-specific knowledge or physics-informed models could enhance robustness.

The proposed LAIML-MFRPPPA model outperforms state-of-the-art methods by achieving the lowest prediction errors while maintaining high interpretability and computational efficiency. Unlike deep models such as polyBERT and GNN, which require complex architectures, our approach is lightweight, easier to deploy in industrial settings, and offers real-time applicability with strong predictive accuracy.

## 5. Conclusions

This study presents the LAIML-MFRPPPA model, which focuses on developing accurate and reliable predictive models for melt flow rate (MFR) using advanced machine learning techniques. The modeling framework begins with min–max normalization to scale the input features into a consistent range, followed by the implementation of ensemble models—namely the kernel extreme learning machine (KELM) and random vector functional link (RVFL). These models are further optimized using the pelican optimization algorithm (POA) to enhance predictive accuracy. A benchmark dataset consisting of 1044 polymer samples was used for experimental evaluation, incorporating six key input features relevant to polymer processing. The results demonstrate that the proposed LAIML-MFRPPPA model achieved superior performance compared to traditional and deep learning baselines, with an R^2^ of 0.965, a mean absolute percentage error (MAPE) of 3.4%, and a root mean square error (RMSE) of 0.12. In addition, SHAP-based sensitivity analysis confirmed the model’s interpretability by identifying key input features influencing MFR prediction. The ensemble framework not only delivers high prediction accuracy, but also ensures robustness and generalizability through cross-validation and optimized hyperparameter selection. Its lightweight structure and compatibility with industrial platforms such as Python (3.13.7) and TensorFlow further support practical deployment in real-time manufacturing settings. Overall, the LAIML-MFRPPPA model offers a powerful and scalable solution for predictive quality monitoring in polymer production environments.

## Figures and Tables

**Table 1:** Input feature descriptions.

Feature Name	Variable	Unit	Physical Meaning
Reactor temperature	Temp	°C	Affects viscosity; higher temp reduces melt resistance
Hydrogen to propylene ratio	H_2_/C_3_ = (H2R)	Ratio	Controls molecular weight via chain termination
Reactor pressure	Pressure	bar	Affects molecular mobility and polymer weight distribution
Reactor bed level	Level	meters	Indicates polymer residence and reaction consistency
Ethylene flow rate	C_2_ = Flow	kg/hr	Co-monomer affecting softness and flow
Catalyst feed rate	Cat Feed	kg/hr	Controls reaction rate and structural formation
Propylene feed rate	C_3_ = Feed	kg/hr	Main monomer source; determines base chain properties
Melt flow rate	MFR	g/10 min	Target variable; evaluates processability and polymer quality

**Table 2:** Classifier result of the LAIML-MFRPPPA model below various metrics.

Metrics	Training Set	Testing Set
MSE	0.0083	0.0079
RMSLE	0.0713	0.0706
MAE	0.0659	0.0673
MAPE	0.3611	0.3697

**Table 3:** Comparative analysis of the LAIML-MFRPPPA model with existing methods.

Methods	MSE	MAE	MAPE
Linear regression	0.0521	0.1087	0.1091
Support vector regression	0.0446	0.1024	0.1027
Decision tree	0.0393	0.0966	0.1387
Adaboost	0.034	0.091	0.1570
Bonett	0.0285	0.0851	0.1747
Levene	0.0219	0.0773	0.2537
Random forest	0.0147	0.0716	0.3137
LAIML-MFRPPPA	0.0079	0.0659	0.3697

**Table 4:** Comparison of deep learning models.

Model	MSE	MAE	MAPE
polyBERT	0.0091	0.0684	0.3749
GNN	0.0102	0.0711	0.3902
LSTM	0.0086	0.0679	0.3674
LAIML-MFRPPPA	0.0079	0.0659	0.3697

## Data Availability

The original contributions presented in this study are included in the article. Further inquiries can be directed to the corresponding authors.
